# Modification Approaches of Polyphenylene Oxide Membranes to Enhance Nanofiltration Performance

**DOI:** 10.3390/membranes13050534

**Published:** 2023-05-21

**Authors:** Mariia Dmitrenko, Xeniya Sushkova, Anastasia Chepeleva, Vladislav Liamin, Olga Mikhailovskaya, Anna Kuzminova, Konstantin Semenov, Sergey Ermakov, Anastasia Penkova

**Affiliations:** 1 St. Petersburg State University, 7/9 Universitetskaya nab., Saint Petersburg 199034, Russia; st111000@spbu.student.ru (X.S.); chepeleva1999@yandex.ru (A.C.); lyamin.vlad.322@gmail.com (V.L.); st113220@student.spbu.ru (O.M.); a.kuzminova@spbu.ru (A.K.); s.ermakov@spbu.ru (S.E.); a.penkova@spbu.ru (A.P.); 2 Pavlov First Saint Petersburg State Medical University, L’va Tolstogo ulitsa 6–8, Saint Petersburg 197022, Russia; semenov1986@yandex.ru

**Keywords:** polyphenylene oxide, mixed matrix membrane, graphene oxide, layer-by-layer technique, polyelectrolytes, nanofiltration, dye

## Abstract

Presently, water pollution poses a serious threat to the environment; the removal of organic pollutants from resources, especially dyes, is very important. Nanofiltration (NF) is a promising membrane method to carry out this task. In the present work, advanced supported poly(2,6-dimethyl-1,4-phenylene oxide) (PPO) membranes were developed for NF of anionic dyes using bulk (the introduction of graphene oxide (GO) into the polymer matrix) and surface (the deposition of polyelectrolyte (PEL) layers by layer-by-layer (LbL) technique) modifications. The effect of PEL combinations (polydiallyldimethylammonium chloride/polyacrylic acid (PAA), polyethyleneimine (PEI)/PAA, and polyallylamine hydrochloride/PAA) and the number of PEL bilayers deposited by LbL method on properties of PPO-based membranes were studied by scanning electron microscopy (SEM), atomic force microscopy (AFM), and contact angle measurements. Membranes were evaluated in NF of food dye solutions in ethanol (Sunset yellow (SY), Congo red (CR), and Alphazurine (AZ)). The supported PPO membrane, modified with 0.7 wt.% GO and three PEI/PAA bilayers, exhibited optimal transport characteristics: ethanol, SY, CR, and AZ solutions permeability of 0.58, 0.57, 0.50, and 0.44 kg/(m^2^h atm), respectively, with a high level of rejection coefficients—58% for SY, 63% for CR, and 58% for AZ. It was shown that the combined use of bulk and surface modifications significantly improved the characteristics of the PPO membrane in NF of dyes.

## 1. Introduction

Currently, water pollution poses a serious threat to the environment, and the removal of organic and toxic pollutants from resources attracts a great amount of attention from scientists [1]. The textile and food industries are the main sources of pollution, of which significantly affects the quality of available water resources due to the discharge of dyes. Dyes (more than 100,000 commercially available) are complex organic molecules widely used in industries of printing, plastics, paper, textile, food, etc. [2,3,4,5]. Most dyes are not amenable to traditional methods of purification, thereby accumulating in the environment due to their high degree of resistance to temperature, light, biodegradation, and detergents [6]. Pressure-driven membrane process nanofiltration (NF) is one of the most promising alternatives for resource purification from dyes, as it relates to sustainable processes [7]. NF is a pressure-driven membrane process between reverse osmosis (RO) and ultrafiltration (UF), and is used for removing solutes with a molecular weight in the range of 200–1000 g/mol [8,9]. A current area of intense research is the extension of NF to the separation of molecules in organic solvents, which is called organic solvent NF (OSN) [8,10] and has a great potential in industries (from refining to chemical and pharmaceutical synthesis) [11]. Both porous and non-porous (composite or supported) membranes are used for OSN [12] of the most common solvents and solutes [11], and various modification approaches are used for the development of NF membranes to increase filtration efficiency. Scientists are also actively developing mathematical models of NF to better understand transport mechanisms as well as to predict separation characteristics. For example, models based on the one-dimensional Nernst–Planck equation coupled with electroneutrality, zero current, and Donnan equilibrium conditions as well as two-dimensional Nernst–Planck, Poisson, and Navier–Stokes equations, a novel equation for salt flux obtained from the full solution-friction (SF) theory, were applied to investigate theoretically the pressure-driven transport of electrolytes through membranes [13,14].

In this work, it was decided to investigate supported membranes based on an aromatic glassy polymer poly(2,6-dimethyl-1,4-phenylene oxide) (PPO) with a high thermal and mechanical stability as well as a resistance to chemical agents [15] for NF of food dye solutions in ethanol. This polymer is actively applied as a membrane material for diffusion membrane processes (gas separation [16,17] and pervaporation [18,19,20,21]). Based on a literature review, it was found that supported membranes based on pristine (unfunctionalized) PPO have not been yet studied for NF. Its derivatives (for example, brominated and sulfonated PPO) were mainly used for NF of different electrolytes [22,23,24,25,26]. Only membranes from brominated PPO (BPPO) were developed for OSN (namely, for the rejection of dyes such as Bengal rose, Safranine T, Alizarin yellow GG, Eriochome black T, Crystal violet, Bromophenol blue, and Coomassie brilliant blue R250) [27]. Thus, the aim of this work was to investigate the developed supported PPO membrane in NF of dyes and to enhance its performance using various modification approaches: bulk (the introduction of graphene oxide (GO) into the polymer matrix) and surface (the deposition of polyelectrolytes (PEL) with layer-by-layer (LbL) technique) modifications.

The creation of a mixed matrix membrane is a promising modification method, consisting in the introduction of an organic/inorganic component into the polymer matrix. It allows a direct and flexible changing of the membrane characteristics, combining advantages of both components. In the present work, the improvement of NF PPO membrane properties was achieved by the modification with GO due to its unique structure, good dispersion in the PPO matrix, and functional (oxygen-containing) groups. The modification with GO also provides a perspective on the improvement of NF membranes for the rejection of negatively charged molecules [28]. Our previous work [21] demonstrated the use of GO as a modifier for the pervaporation PPO membrane, which led to an increased membrane permeation flux, maintaining a high level of selectivity in the dehydration of ethylene glycol. 

The deposition of nano-sized PEL layers by LbL assembly is a surface modification method, which can effectively adjust the membrane performance to obtain the tailored permeability and/or selectivity [29]. The coating of PEL layers on the membrane surface leads to definite surface charge and high hydrophilicity, resulting in a strong affinity for polar molecules, which are attractive for their use in surface membrane modification. To achieve the tailored membrane performance, the characteristics of a surface-modified membrane may be varied by the choice of PEL pair, number of deposited layer, PEL charge density, pH, etc. [30,31]. 

Thus, the aim of this study was to develop PPO membranes with improved characteristics by the use of bulk (the introduction of GO into the polymer matrix) and surface (the deposition of PEL by LbL technique) modifications for enhanced NF of food anionic dyes. Four PEL (three cationic–polydiallyldimethylammonium chloride, polyethyleneimine, polyallylamine hydrochloride and one anionic–polyacrylic acid) with different charge densities of a PEL pair were used to carry out the surface modification of PPO membranes. The obtained membranes were investigated by various analysis methods such as scanning electron microscopy (SEM), atomic force microscopy (AFM), and contact angle measurements. The transport properties of membranes were evaluated in NF of ethanol and solutions of anionic food dyes (Sunset yellow (SY, E110), Congo red (CR, E129), and Alphazurine (AZ, E133)) with various molecular weights. The stability of the PEL layer on the membrane surface was confirmed after NF by data of FTIR, SEM, AFM and contact angle measurements.

## 2. Materials and Methods

### 2.1. Materials

Poly(2,6-dimethyl-1,4-phenylene oxide) (PPO, CAS Number: 25134-01-4, 1.06 g/mL, Sigma-Aldrich, St. Petersburg, Russia) was applied as a membrane matrix. Graphene oxide (GO, Fullerene Technologies, St. Petersburg, Russia) was applied as a modifier for the volume (bulk) modification of the PPO membrane. For the preparation of the supported PPO membranes, commercial membrane MFFC (Vladipor, Vladimir, Russia) from fluoroplast F42L was applied as a porous support. For surface modification with polyelectrolytes (PEL) of the PPO membranes by layer-by-layer (LbL) technique, polyacrylic acid (PAA, CAS Number: 9003-01-4, Mw~100,000, 35 wt.% in H_2_O, Sigma-Aldrich, St. Petersburg, Russia), polyallylamine hydrochloride (PAH, CAS Number: 71550-12-4, Mw~150,000, 40 wt.% in H_2_O, Polysciences Europe GmbH, Hirschberg an der Bergstrasse, Germany), polyethyleneimine (PEI, CAS Number: 9002-98-6, Mw~25,000, 50 wt.% in H_2_O, Acros Organics, Moscow, Russia), and polydiallyldimethylammonium chloride (PDADMAC, CAS Number: 26062-79-3, Mw~200,000-350,000, 20 wt.% in H_2_O, Sigma-Aldrich, St. Petersburg, Russia) were used. Characteristics of PEL are presented in Table 1. Chloroform (CHCl_3_, CAS Number: 67-66-3, 99.1 wt.%, Vekton, St. Petersburg, Russia) was used without additional treatment.

### 2.2. Membrane Preparation

#### 2.2.1. Supported Membranes

The PPO/GO composites were prepared by solid-phase method: the determined amount of PPO was ground with the GO (0.5, 0.7, 1, and 1.5 wt.% with the respect to the PPO weight), followed by dissolution in chloroform to obtain a 5 wt.% solution. Supported membranes were prepared by physical adsorption method: the 5 wt.% PPO solution in chloroform and PPO/GO composite were deposited onto a porous commercial MFFC membrane for the formation of a thin dense selective layer, followed by solvent evaporation at ambient temperature for 24 h [21].

#### 2.2.2. Surface Modification with PEL by Layer-by-Layer Technique

**Figure 1:**
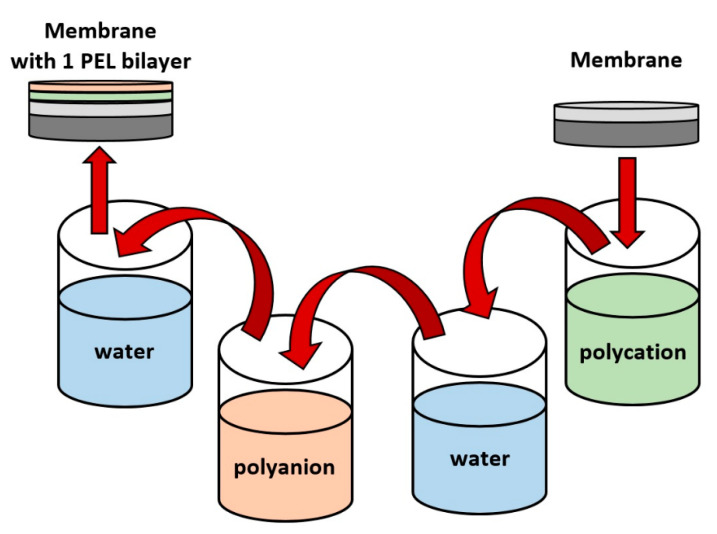
Layer-by-layer (LbL) deposition scheme of one PEL bilayer.

Surface modification of the supported membranes with PEL by LbL assembly was carried out using a Xdip-MV1 robotic coating immersion system (PROMENERGOLAB Ltd., Moscow, Russia) with the application of the PAA polyanion, and PAH, PEI, and PDADMAC polycations (10^−2^ mol/L in water). To prevent the exposure of the porous substrate to PEL, the supported membrane was fixed with silica gel on a Teflon plate (selective PPO layer oriented outward) and immersed alternately in PEL solutions for 10 min with water washing between the immersions [32]. The pH of the PAA and PAH solutions was adjusted to 6.5 due to maximum ionization of these weak polyelectrolytes being at this pH [31]. The polycations (PAH, PEI, and PDADMAC) were deposited initially. The membrane was washed with water several times after the deposition of the polycations. Polyanion PAA was deposited next, also followed by water rinsing. This completed the formation of one PEL bilayer on the membrane surface. The LbL modification procedure for the deposition of 1 PEL bilayer is schematically presented in Figure 1.

### 2.3. Nanofiltration (NF)

**Figure 2:**
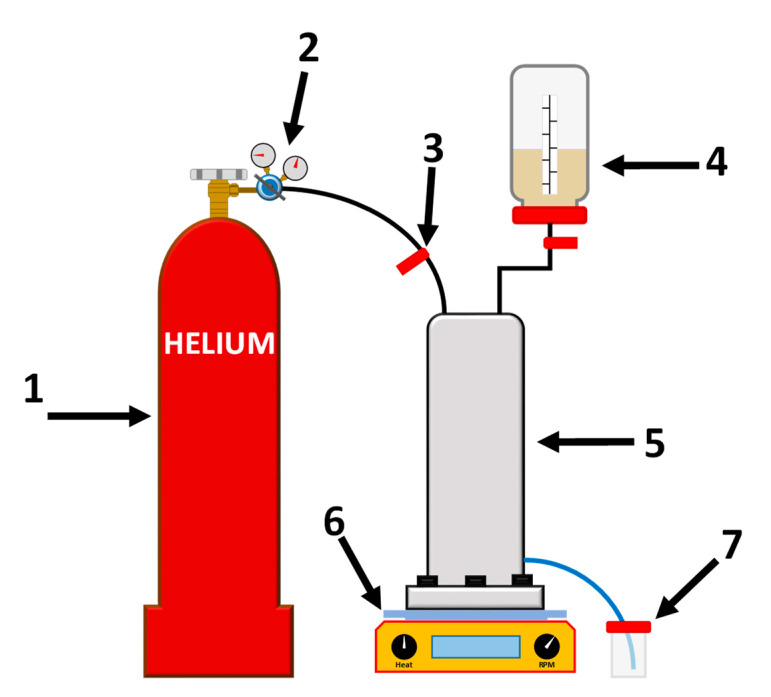
The scheme of the NF setup: 1—gas cylinder, 2—pressure regulator, 3—pressure valve, 4—feed tank, 5—membrane cell, 6—magnetic stirrer, 7—tank for permeate.

The developed supported membranes were evaluated in NF of food dye (Sunset yellow (SY, E110), Congo red (CR, E129), and Alphazurine (AZ, E133)) solutions in ethanol (10 mg/L), using a dead-end cell with 0.2·10^−2^ m^2^ effective area under constant stirring at ambient temperature [33]. The scheme of the NF is presented in Figure 2 [33]. NF experiments were carried out for at least a week for each membrane; every day, the membrane was tested in NF of ethanol and dye (SY, CR, and AZ) solutions, alternating them each time (namely, ethanol, SY solution, ethanol, CR solution, ethanol, AZ solution, and ethanol). Ethanol was passed between the dye solutions to confirm the stability of membrane properties. In an attempt to avoid the effects of concentration polarization, feeds were actively stirred, and ethanol was passed after each dye solution. Concentration polarization significantly decreases for dilute solutions (0.01 g/L) [34]. Within a week, the data obtained were averaged, and the membranes demonstrated the stability of the transport characteristics (the obtained average accuracies were as follows: ±5% for permeability, and ±2% for rejection coefficient).

The content of dyes in the feed and permeate was studied using a spectrophotometer PE-5400UV (EKROSKHIM, St. Petersburg, Russia), at 483 nm for Sunset yellow (E110), 505 nm for Congo red (E129), and 628 nm for Alphazurine (E133), corresponding to the absorbance maximum. The structure of dyes is presented in Table 2.

Permeability was calculated according to the following equation [35]:

(1) $$L=\frac{J}{\Delta P}=\frac{m}{A\cdot t\cdot \Delta P},$$

where m is the weight of permeate (kg), t is permeate collection time (h), A is effective surface area of membrane (m^2^), and ΔP is transmembrane pressure (atm).

The rejection coefficient of dyes was calculated according to the following equation:

(2) $$R=\left(1-\frac{C_{\mathrm{perm}}}{C_{\mathrm{feed}}}\right)\cdot \;100\%,$$

where C_perm_ and C_feed_ are the concentration of dyes in the permeate and the feed, respectively.

### 2.4. Fourier-Transform Infrared Spectroscopy (FTIR)

The structure of the upper selective layer of supported membranes was investigated using an IRAffinity-1S spectrometer (Shimadzu, St. Petersburg, Russia) with an attenuated total reflectance (ATR) accessory (PIKE Technologies, St. Petersburg, Russia) at ambient temperature in the range of 500–4000 cm^−1^.

### 2.5. Scanning Electron Microscopy (SEM)

The cross-sectional and surface morphology of supported membranes was studied using a Zeiss Merlin SEM (Carl Zeiss SMT, Oberhochen, Germany). The membrane cross-section was obtained by cleaving in liquid nitrogen.

### 2.6. Atomic Force Microscopy (AFM)

The surface topography of the developed membranes was investigated using an NT-MDT NTegra Maximus atomic force microscope (NT-MDT Spectrum Instruments, Moscow, Russia) in the tapping mode using standard silicon cantilevers with 15 N·m^−1^ rigidity.

### 2.7. Contact Angle Measurements

To evaluate the hydrophilic–hydrophobic surface properties, water contact angles were measured using a Goniometer LK-1 device (NPK Open Science Ltd., Krasnogorsk, Russia) by the sessile drop method. The “DropShape” software (the Laboratory of Mathematical Methods of Image Processing, Lomonosov Moscow State University, Moscow, Russia) was used to analyze results. At least three different locations were measured for each membrane, and the average contact angles were presented.

## 3. Results

This section is divided into three main parts. Section 3.1 is dedicated to the investigation of NF performance of the PPO-based membranes, including subsections devoted to the investigation of PPO membranes with bulk modification by GO (Section 3.1.1), with surface modification by various PEL composition and bilayer numbers (Section 3.1.2), and PPO membranes modified with GO and PEL (Section 3.1.3). The characterization of the developed supported membranes by various analysis methods is presented in Section 3.2. In Section 3.3, the comparison of NF performance of PPO-based membranes with membranes described in the literature is presented.

### 3.1. Nanofiltration Performance

#### 3.1.1. Study of GO Effect

**Figure 3:**
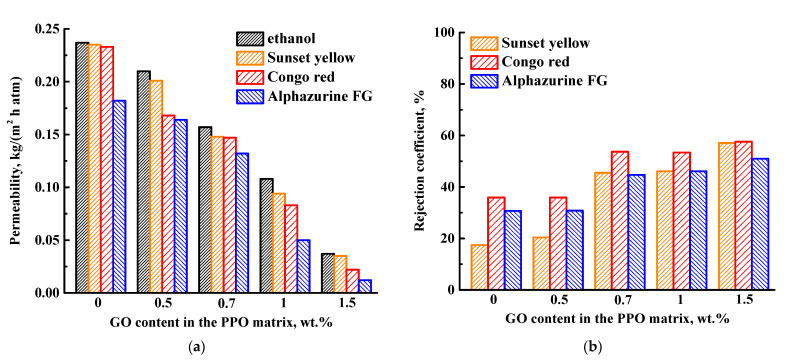
(**a**) Permeability and (**b**) rejection coefficients of the supported PPO and PPO/GO membranes in NF of ethanol and dye solutions.

To study the effect of volume modification with GO, the supported PPO and PPO/GO (0.5–1.5 wt.%) membranes were tested in the NF of ethanol and solutions of anionic dyes (SY, CR, and AZ) with different molecular weights (Figure 3).

For all membranes, ethanol permeability was higher compared to the permeability of dye solutions due to the membrane contamination by dyes, causing membrane blocking [36]. The permeability of ethanol, SY, and CR solutions for the pristine PPO membrane was 0.237, 0.235, and 0.233 kg/(m^2^h atm), respectively, while the permeability of AZ decreased down to 0.182 kg/(m^2^h atm). It may be explained by another interaction type between the AZ and membrane selective layer: hydrophobic or hydrogen bonding, resulting in membrane blocking [36]. The introduction of GO into the PPO matrix led to a decrease in the permeability of the ethanol and dye solutions, compared to the pristine PPO membrane (Figure 3a), which intensified with the increase in GO content. This behavior can be explained by the cross-linking effect of GO on the polymer matrix [37], and the formation of GO agglomerates in the membrane matrix [38], which hindered the mass transfer of permeate components [39]. The interaction between PPO and GO was confirmed in the previous work [21].

The rejection coefficient of the membrane in NF may be explained by several mechanisms (molecular sieves, differences in diffusion and solubility, and the Donnan effect) [40]. The separation of dye molecules is mainly conditioned to their molecular weight (sieving) and electrostatic (charge) effects [41]. The increase of the molecular weight of dyes (Table 2) resulted in an increase of membrane selectivity (rejection coefficient) (Figure 3b) [10,36], except for CR dye. The highest rejection coefficient of CR for all PPO and PPO/GO membranes may be due to larger aggregates of this dye at the pH of ethanol (~6.8), as the pH solution influences the charge and structure of the CR dye molecules [42]. The modification of the PPO membrane with GO particles led to the increase of dye rejection coefficients, which increased with the increase of GO content in the PPO membrane (Figure 3b). The introduction of GO increased the rejection ability of the anionic dyes for the modified PPO/GO membranes because it led to the formation of a more negatively charged membrane surface, causing electrostatic repulsion between the dye molecules and membrane surface [40,41]. The same effect was demonstrated for NF composite membranes from polyethersulfone with graphene oxide and sulfonated graphene oxide [43]. The optimal NF performance was observed for the PPO membrane modified with 0.7 wt.% GO: the optimal ratio of permeability (0.148 kg/(m^2^h atm) for ethanol, and 0.157, 0.147, 0.132 kg/(m^2^h atm) for SY, CR, and AZ solutions, respectively) and increased dye rejection coefficients (46, 54, and 45%, for SY, CR, and AZ, respectively). Thus, this membrane was chosen for further surface modification with polyelectrolytes (PEI/PAA) to increase permeability (Section 3.1.3).

#### 3.1.2. Study of the Effect of PEL Composition and Bilayer Number

**Figure 4:**
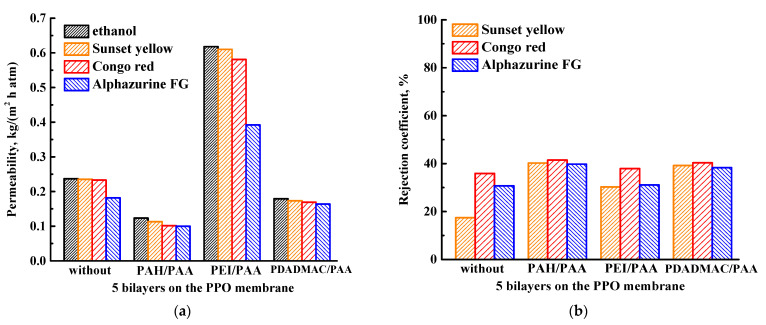
(**a**) Ethanol and dye solution permeability and (**b**) rejection coefficients for the PPO membranes with the five deposited PEL bilayers.

Currently, there is no guideline for selecting commercial PEL according to the separation type being studied. In this work, only four PEL (three cationic (PDADMAC, PEI, and PAH) and one anionic (PAA)) with different charge densities of a PEL pair (Table 1) were used to carry out surface modification of the PPO membrane by LbL technique. In organic media, the charge density of a PEL pair plays the role of a physical cross-linking agent, affecting mass transfer through the surface modified membrane [31]. A total of five bilayers of PDADMAC/PAA, PAH/PAA, and PEI/PAA were deposited onto the PPO membrane and tested in the NF of ethanol and dye (SY, CR, and AZ) solutions (Figure 4).

It was demonstrated that the permeability of membranes surface-modified with five bilayers of PAH/PAA and PDADMAC/PAA decreased compared to the PPO membrane (Figure 4a), due to the high charge density of these PEL pairs (4.3 × 10^−3^ and 6.0 × 10^−3^ for PDADMAC/PAA and PAH/PAA, Table 1). The highest permeability was observed for the PPO/five bilayers of PEI/PAA membrane compared to all membranes. This may be due to the low charge density of this PEL pair (Table 1), the highest surface roughness and hydrophilicity for this membrane among surface-modified membranes (confirmed by AFM and contact angle data, presented below). Ethanol can pass through the membrane with a more effective surface area (due to high surface roughness) under pressure, forming special pathways for the transport of molecules through the relatively soft PEI/PAA layer (due to the low charge density of the PEL pair), resulting in the improved permeability [43]. The deposition of five PEL bilayers onto the PPO membrane led to the increased dye rejection coefficients (Figure 4b), attributed to the upper negatively charged PAA layer and its functional carboxylic acid groups [44]. Electrostatic repulsion between like charges (anionic PAA and dyes) is assumed [45]. However, surface modification with PEL resulted in a slight increase in dye rejection (no more than 42%). Thus, the PEI/PAA pair was chosen for surface modification of the PPO membrane in order to increase permeability in NF.

**Figure 5:**
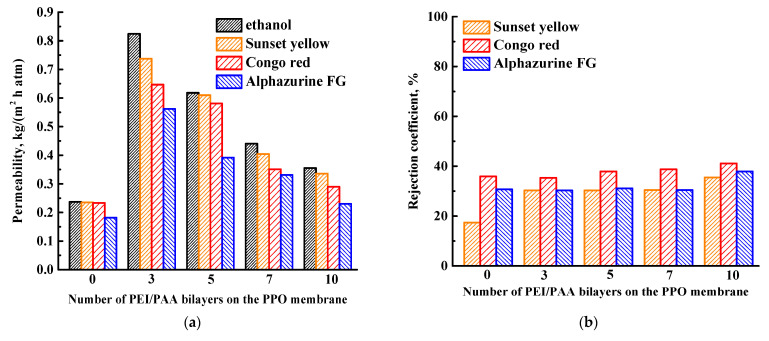
(**a**) Ethanol and dye solution permeability and (**b**) rejection coefficients for the PPO membranes with various numbers of PEI/PAA bilayers deposited.

In the second step, the influence of the number of deposited bilayers of PEI/PAA (3-10 bilayers) on the performance of the surface-modified PPO membrane in the NF of ethanol and dye (SY, CR, and AZ) solutions was investigated (Figure 5).

It was shown that an increase from three to ten of the deposited number of PEI/PAA bilayers on the PPO membrane led the decrease of permeability and slight increase of dye rejection coefficients (Figure 5a,b), due to formation of a thicker upper PEL layer [43]. The PPO/3 bilayers of PEI/PAA membrane had the highest permeability (0.824, 0.737, 0.647, and 0.562 kg/(m^2^h atm) for ethanol, SY, CR, and AZ, respectively). The further improvement of membrane performance was carried out by a combination of bulk (the introduction of GO into the PPO matrix) and surface (coating with the optimal three PEI/PAA bilayers on the membrane surface) modifications [46].

#### 3.1.3. Study of Membranes Modified with GO and PEL

**Figure 6:**
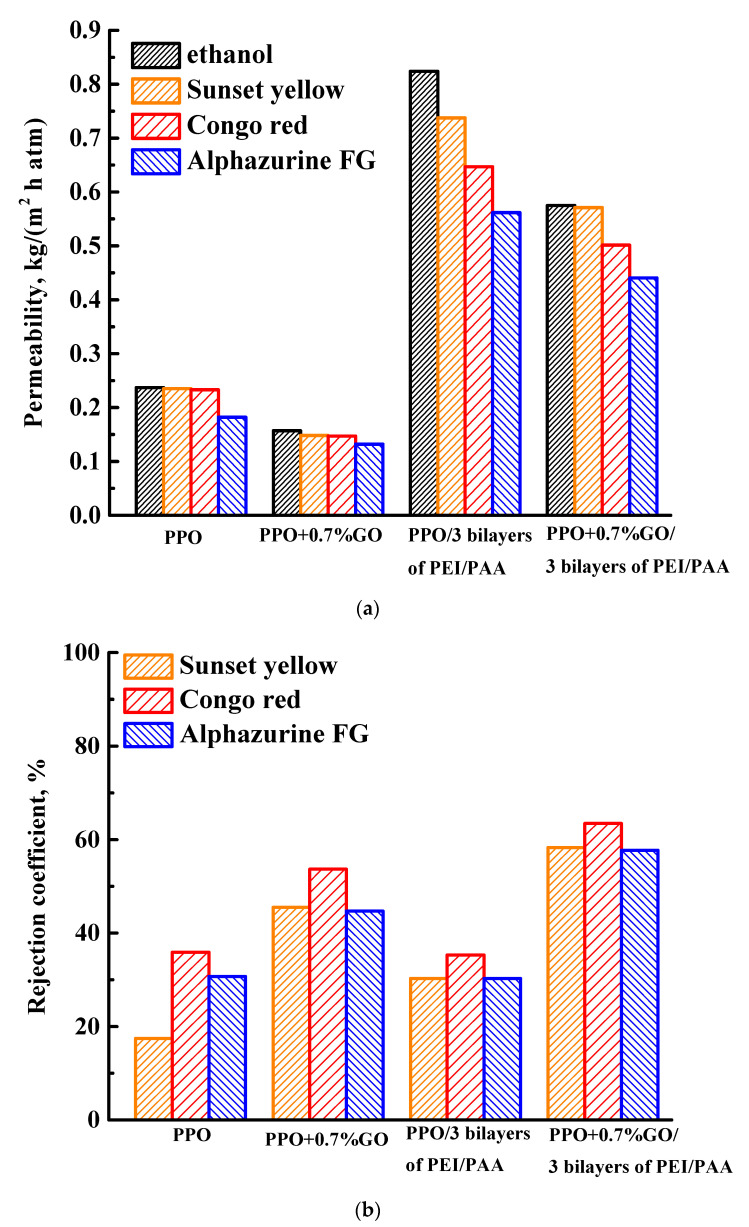
(**a**) Ethanol and dye solution permeability and (**b**) rejection coefficients for the PPO and PPO/GO membranes without/with the three deposited bilayers of PEI/PAA.

To increase permeability, the PPO + 0.7% GO membrane was surface-modified with a deposition of three bilayers of PEI/PAA and also tested in the NF of ethanol and dye (SY, CR, and AZ) solutions (Figure 6). The data of the PPO, PPO + 0.7% GO, and PPO/3 bilayers of PEI/PAA membranes were also presented in Figure 6 for comparison.

It was demonstrated that the use of bulk (the introduction of 0.7 wt.% GO into the PPO matrix) and surface modification (the deposition of three bilayers of PEI/PAA by LbL assembly) mutually was shown to result in optimal membrane performance. Specifically, the PPO + 0.7% GO/3 bilayers of PEI/PAA membrane had increased permeability 2.4 times (with an ethanol, SY, CR, and AZ solution permeability of 0.58, 0.57, 0.50, and 0.44 kg/(m^2^h atm), respectively) and increased dye rejection coefficients by 41, 28, and 27% (58% for SY, 63% for CR, and 58% for AZ) compared to the pristine PPO membrane. Based on previous NF data (Figure 3, Figure 4 and Figure 5), the introduction of GO into the PPO matrix and the top PAA layer (forming three PEI/PAA bilayers) results in an improved rejection ability due to the GO oxygen-containing groups and negative charge of PAA. The application of three soft PEI/PAA bilayers with a low charge density (Table 1) on the membrane surface by LBL method promotes the formation of transport channels for the transfer of ethanol in the upper PEL layer, as well as improved surface roughness and hydrophilicity of the membrane (confirmed by AFM and contact angle data, presented below), resulting in an increase in permeability. Thus, the PPO membrane modified with 0.7 wt.% GO and with the deposition of three PEI/PAA bilayers (PPO + 0.7% GO/3 bilayers of PEI/PAA) had the optimal transport properties in NF of anionic food dye solutions.

### 3.2. Membrane Characterization

**Figure 7:**
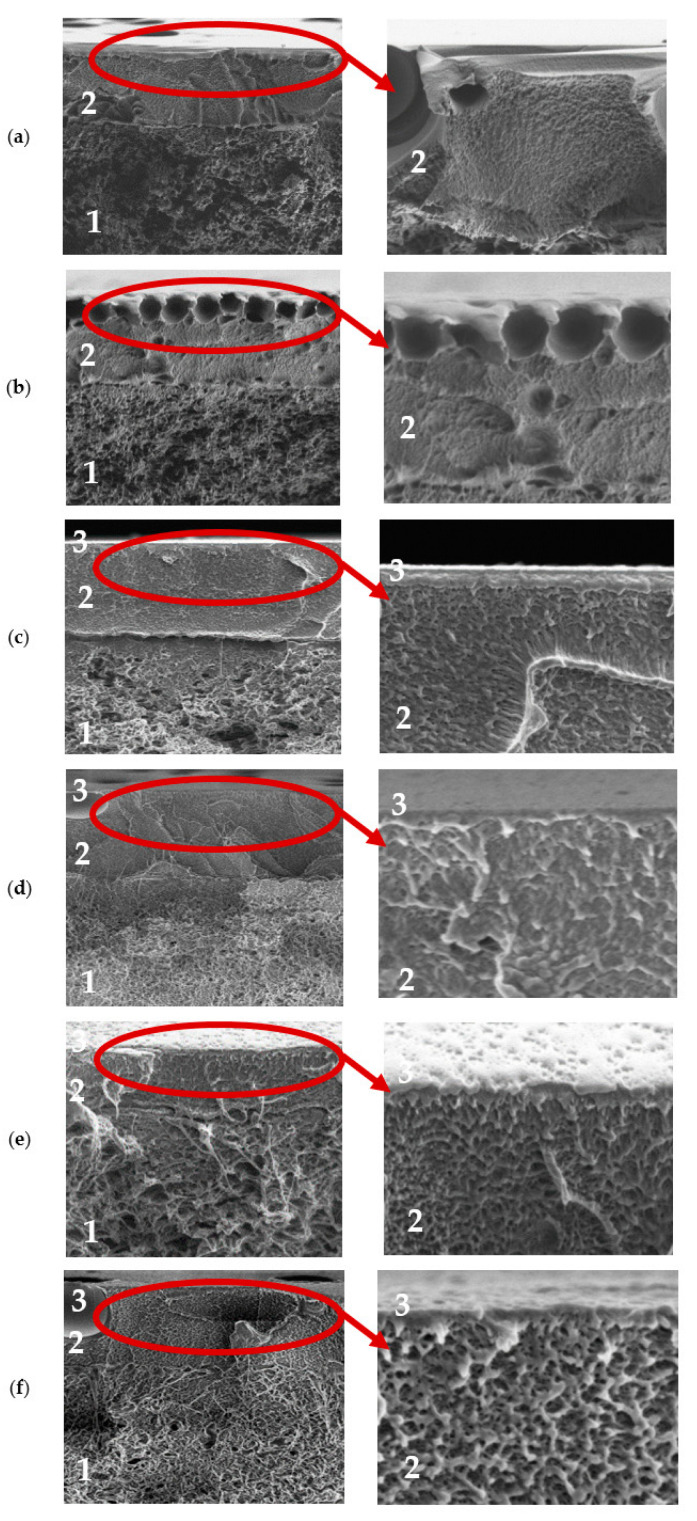
SEM cross-sectional micrographs with magnification 10 kX and 25 kX of (**a**) PPO, (**b**) PPO + 0.7% GO, (**c**) PPO/5 bilayers of PDADMAC/PAA, (**d**) PPO/5 bilayers of PAH/PAA, (**e**) PPO/5 bilayers of PEI/PAA, and (**f**) PPO + 0.7% GO/3 bilayers of PEI/PAA membranes. 1—region of porous MFFC support, 2—region of the dense selective layer, 3—region of the PEL layer.

**Figure 8:**
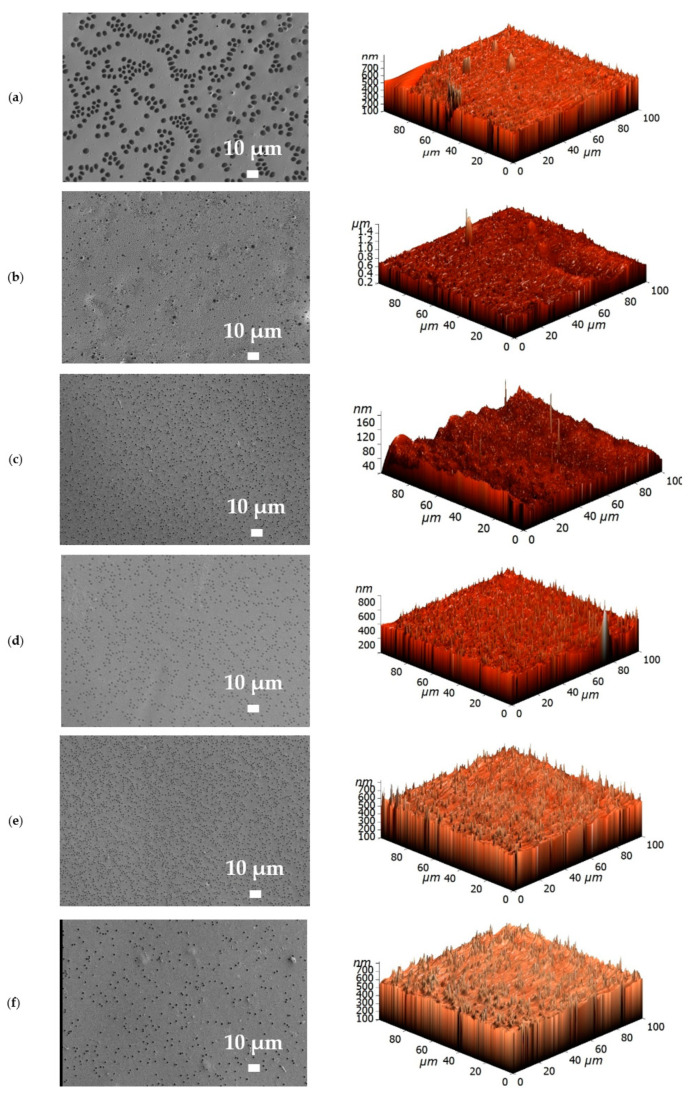
Surface SEM micrographs and AFM images of (**a**) PPO, (**b**) PPO + 0.7% GO, (**c**) PPO/5 bilayers of PDADMAC/PAA, (**d**) PPO/5 bilayers of PAH/PAA, (**e**) PPO/5 bilayers of PEI/PAA, and (**f**) PPO + 0.7% GO/3 bilayers of PEI/PAA membranes.

To confirm the formation of the PEL layer and to evaluate surface parameters, supported membranes were studied by SEM, AFM, and contact angle measurements. The SEM micrographs and AFM images of the supported PPO and PPO/GO (0.7%) membranes without/with PEL modification are presented in Figure 7 and Figure 8.

For the supported PPO and PPO + 0.7% GO membranes, the cross-sectional SEM micrographs clearly demonstrate only two regions (Figure 7a,b): (1) the porous MFFC substrate, and (2) a thin dense selective layer based on the PPO and PPO/GO (0.7%) composite with a thickness of 3 ± 0.2 µm, as was also obtained in the previous work [21]. These membranes also have rounded non-perforating cavities on the surface of the thin selective layer (confirmed by SEM surface (Figure 8) and cross-sectional micrographs), which have previously been confirmed in [21,47,48]. For surface-modified membranes, there are three regions: (1) the porous MFFC substrate, (2) a thin dense selective PPO-based layer, and (3) the thinnest PEL layer with a thickness of 60 ± 10 nm (Figure 7c–f). A continuous and uniform adhesion of the thin dense PPO-based layer to the surface of the porous MFFC substrate, as well as of PEL layers to the surface of the thin dense PPO-based layer, was also observed.

The surface SEM micrographs of PPO and PPO + 0.7% GO membranes (Figure 8a,b) were also in agreement with the previously obtained data [21]; when GO is introduced, the number of cavities on the membrane surface increased, while their size decreased. The deposition of the PEL layer on the surface of PPO and PPO + 0.7% GO membranes led to their partial filling (Figure 8c–f). The surface of the supported membranes had a nodule structure, confirmed by AFM data (Figure 8). Based on AFM images, the surface parameters in terms of average (Ra) and root-mean-square roughness (Rq) were evaluated and are presented in Table 3. To assess the hydrophilic—hydrophobic balance of the membrane surface, the water contact angle of water was also measured (Table 3).

It was found that the introduction of 0.7 wt.% GO into the PPO membrane led to the increase of surface roughness parameters due to the formation of a larger number of cavities on the membrane surface, and surface hydrophilization due to the migration of hydrophilic (oxygen-containing) GO groups to the top of the membrane surface [21]. The LbL deposition of PEL bilayers (PDADMAC/PAA, PAH/PAA, PEI/PAA) onto the PPO membrane led to the slight increase of surface roughness parameters (not more than 5 nm). The PPO + 0.7% GO membrane with three deposited bilayers of PEI/PAA had a lower surface roughness compared to the pristine PPO + 0.7% GO membrane. This may be explained by the formation of a 60 nm thick PEL layer covering all irregularities of membrane surface [46]. The deposition of PEL layers onto the PPO membrane led to a decrease of the water contact angle (surface hydrophilization), because of the hydrophilic PEL nature. The PEL charge density affects the contact angle of surface-modified membranes, regardless of the sign of the surface charge [49]. The water contact angle values decreased for surface-modified PPO membranes when five bilayers were applied: PDADMAC/PAA > PAH/PAA > PEI/PAA. Despite the fact that PAA was applied last on top of all membranes, the slight difference in values was partly due to the anionic or cationic nature of the PEL charge, but mainly due to differences in the intrinsic hydrophobicity of PEL molecules [49]. The contact angles of PPO/5 bilayers of PEI/PAA and PPO + 0.7% GO/3 bilayers of PEI/PAA membranes were equal to 71° because of a 60 nm thick PEL layer on the membrane surface with the upper PAA layer. It is consistent with the properties of pristine PAA [45].

To confirm the stability of the PEL layer, the PPO + 0.7% GO/3 bilayers of PEI/PAA membrane with optimal properties was studied by FTIR, SEM, AFM, and contact angle measurements after NF.

**Figure 9:**
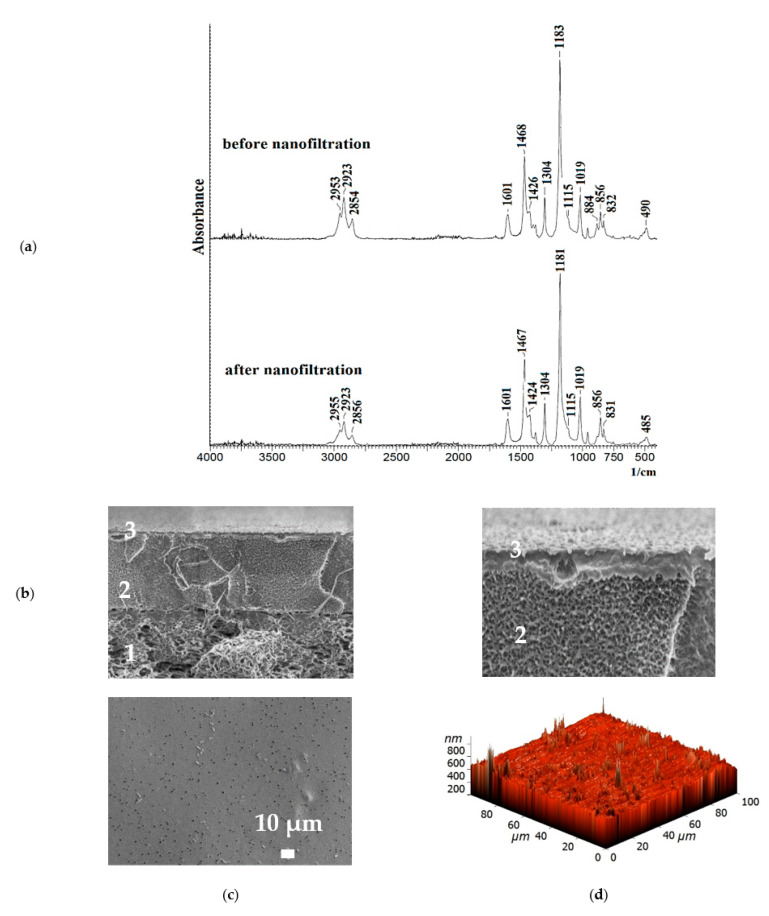
(**a**) FTIR spectra of the PPO + 0.7% GO/3 bilayers of PEI/PAA membrane before and after NF, (**b**) SEM cross-sectional micrograph with magnification 20 kX and 50 kX (1—region of the porous MFFC support, 2—region of the dense selective PPO/GO layer, 3—region of the PEI/PAA layer), (**c**) SEM surface micrograph, and (**d**) AFM surface image of the PPO + 0.7% GO/3 bilayers of PEI/PAA membrane after NF.

It was demonstrated that FTIR spectra of the PPO + 0.7% GO/3 bilayers of PEI/PAA membrane before and after NF experiment were comparatively the same (Figure 9a). The SEM cross-sectional micrograph (Figure 9b) confirmed the maintenance of the PEI/PAA layer on the surface of the membrane that was uniform and did not wash off during the experiment. There was no significant changes on the PPO + 0.7% GO/3 bilayers of PEI/PAA membrane surface after NF; the surface SEM micrograph after NF (Figure 9c) was comparatively the same when compared to the one before the experiment (Figure 8f). Based on AFM image (Figure 9d), the calculated average (Ra) and root-mean-square roughness (Rq) after NF were equal to 24.8 and 50.2 nm, respectively. The PPO + 0.7% GO/3 bilayers of PEI/PAA membrane after NF had a water contact angle of 70°. Thus, the stability and preservation of the PEL layer of PPO + 0.7% GO/3 bilayers of PEI/PAA membrane after the experiment was shown.

### 3.3. Membrane Performance Comparison in Nanofiltration

The performance of membranes described in the literature for NF of anionic dye solutions in ethanol was compared with the developed PPO + 0.7% GO/3 bilayers of PEI/PAA membrane under close experiment conditions, including NF of dyes with similar molecular weights, in terms of permeability and rejection coefficient (Table 4).

It was demonstrated that the developed PPO + 0.7% GO/3 bilayers of PEI/PAA membrane had good performance in NF of anionic dye solutions in ethanol; it had the optimal ratio of permeability and rejection of dyes. However, it is largely inferior to TFCM in performance due to a thicker dense PPO-based layer on a porous substrate. This demonstrated the promising application of the developed PPO-based membranes in the NF of anionic dye solutions.

## 4. Conclusions

In the present work, the advanced supported poly(2,6-dimethyl-1,4-phenylene oxide) (PPO) membranes were developed for NF of anionic dyes using bulk (introduction of graphene oxide (GO) into the polymer matrix) and surface (deposition of polyelectrolyte (PEL) layers by layer-by-layer (LbL) technique) modifications.

Bulk modification of the PPO membrane with GO (0.5–1.5 wt.%) led to the decrease of ethanol and dye solutions permeability and to the increase of rejection coefficients due to a more negatively charged membrane surface caused by functional GO groups. The optimal NF performance was possessed by the PPO membrane modified with 0.7 wt.% GO; it had the optimal level of permeability and dye rejection coefficients.

Surface modification by the deposition of five PEL bilayers onto the PPO membrane surface led to the increased dye rejection coefficients, due to the upper negatively charged PAA layer. The permeability of membranes with five bilayers of PAH/PAA and PDADMAC/PAA decreased compared to the PPO membrane, due to the high charge density of these PEL pairs. While the PPO membrane with five deposited bilayers of PEI/PAA had the highest permeability due to forming facilitated pathways for the transport of molecules through the PEI/PAA layer, which exhibited a relatively soft structure due to its low charge density, the highest surface roughness and hydrophilicity observed among the other studied surface-modified membranes (confirmed by AFM and contact angle data). The effect of the number of PEI/PAA bilayers (3–10 bilayers) on properties of PPO membranes was also studied. The increase from three to ten of the deposited number of PEI/PAA bilayers on the PPO membrane surface led to the decrease of permeability and the slight increase of dye rejection coefficients, due to the formation of a thicker upper PEL layer. The surface modification with three bilayers of PEI/PAA was chosen as optimal, as this membrane had the highest permeability.

The further improvement of membrane performance was carried out by a combination of bulk (the introduction of 0.7 wt.% GO into the PPO matrix) and surface (coating with the optimal three PEI/PAA bilayers on the membrane surface) modifications. This membrane had increased permeability of ethanol and dye solutions 2.4 times, and increased dye rejection coefficients by 41, 28, and 27% (for SY, CR, and AZ, respectively), compared to the pristine PPO membrane in NF. Thus, it was demonstrated that the combined use of bulk and surface modifications significantly improved the characteristics of the PPO membrane in NF of anionic dye solutions.

## Figures and Tables

**Table 1:** Main characteristics of PEL.

	PAA	PDADMAC	PAH	PEI
**Monomer unit**	(C_3_H_4_O_2_)_n_	(C_8_H_16_ClN)_n_	(CH_2_CH(CH_2_NH_2_ · HCl))_n_	(C_22_N_11_H_55_)_n_
**Mw unit, g/mol**	72	161.5	93.5	473
**PEL structure**	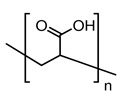	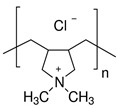	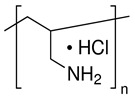	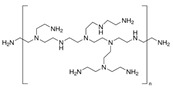
**Mn ^a^**	100,000	200,000–350,000	150,000	60,000
**Charge density by weight (×10^3^) ^b^ [31]**	-	PDADMAC/PAA 4.3	PAH/PAA 6.0	PEI/PAA 1.8

^a^ average molecular weight; ^b^ calculated according to the formula: charge density = 1/∑Mw monomer unit of PEL.

**Table 2:** The structure of dyes.

Dye	Molecular Formula	Structure	Molar Mass, g/mol
Sunset yellow (SY, E110)	C_16_H_10_N_2_Na_2_O_7_S_2_	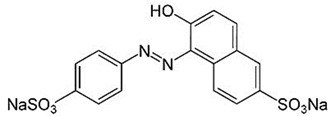	429
Congo red (CR, E129)	C_32_H_22_N_6_Na_2_O_6_S_2_	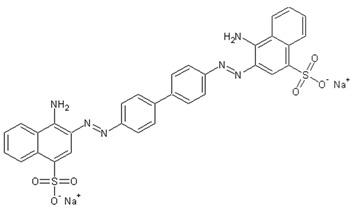	697
Alphazurine (AZ, E133)	C_37_H_34_Na_2_N_2_O_9_S_3_	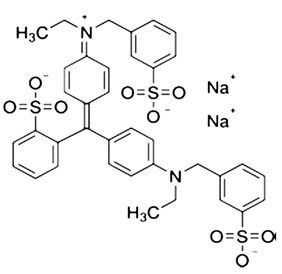	793

**Table 3:** Surface roughness parameters and water contact angle of membranes.

Membrane	Ra, nm	Rq, nm	Contact Angle of Water, °
PPO	24.4	49.1	88 ± 2
PPO + 0.7% GO	44.2	73.0	86 ± 2
PPO/5 bilayers of PDADMAC/PAA	25.1	50.1	75 ± 2
PPO/5 bilayers of PAH/PAA	26.8	50.9	74 ± 2
PPO/5 bilayers of PEI/PAA	28.8	53.4	71 ± 2
PPO + 0.7% GO/3 bilayers of PEI/PAA	26.4	53.1	71 ± 2

**Table 4:** Comparison of transport properties for membranes in NF of anionic dye solutions in ethanol.

Membrane	Ethanol Permeability, kg/(m^2^h atm)	Dye Solution, Concentration	Solution Permeability, kg/(m^2^h atm)	Rejection Coefficient, %	Ref.
PPO + 0.7% GO/3 bilayers of PEI/PAA	0.58	Sunset yellow 10 mg/L	0.57	58	This study
		Congo red 10 mg/L	0.50	63	
		Alphazurine FG 10 mg/L	0.44	58	
PIM-1/MIL-125	-	Alphazurine FG 10 mg/L	0.19	99	[33]
	-	Sunset yellow 10 mg/L	0.19	99	
PIM-1/MIL-140A	-	Alphazurine FG 10 mg/L	0.25	89	
	-	Sunset yellow 10 mg/L	0.23	91	
Cellulose acetate/gold nanoparticles	0.06	Bromothymol Blue	-	82	[50]
Cellophane	0.05	Remazol Brilliant Blue R	-	79	[51]
		Orange II	-	55	
Polyimide (PI)/gold nanoparticles	-	Methyl Orange	0.16	82	[52]
		Bromothymol Blue	2.3	58	[53]
Thin- film nanocomposite membrane (TFCM) polyamide (PA)/polydopamine-HKUST-1_0.6_/polyetherimide	3.6	Congo red 100 mg/L	2.5	93	[54]
		Rose Bengal 100 mg/L	-	91	
		Methyl Orange 100 mg/L	-	80	
TFCM PA-polyether- sulfone/polyvinyl formal	1.6	Orange GII 100 mg/L	-	68	[55]
TFCM PI/hydrolyzed polyacrylonitrile (PAN)	-	Coomassie brilliant blue 100 mg/L	0.55	99	[56]
TFCM PA/octadecylamine (ODA)-functionalized reduced graphene oxide (rGO)/PI	-	Sunset Yellow 20 mg/L	3.6	99	[57]
		Rose Bengal 20 mg/L	3.9	98	

## Data Availability

Data presented in this study are available on request from the corresponding author.
